# Qualitative study reveals perceived barriers and facilitators of preregistration in animal research

**DOI:** 10.1098/rsos.261156

**Published:** 2026-10-28

**Authors:** Cristina Priboi, Simone M. Lalor, Evie Vergauwe, Bernice Elger, Hanno Würbel

**Affiliations:** ^1^ Veterinary Public Health Institute, University of Bern, Bern, Switzerland; ^2^ Institute for Biomedical Ethics, University of Basel, Basel, Switzerland; ^3^ Faculty of Psychology and Educational Sciences, University of Geneva, Geneva, Switzerland

**Keywords:** animal experiments, animal research, open research practices, preregistration, transparency, open science, reproducibility, animal welfare

## Abstract

Preregistration—the prospective registration of hypotheses, methods and analyses plans—is a promising tool in promoting transparency, rigour and reproducibility in science. However, its adoption remains low, particularly in animal research, and little is known about factors influencing its uptake. We explored researchers' experiences and views on preregistration, identifying perceived facilitators and barriers, and gathering suggestions to improve its use. Within a larger cross-sectional mixed-methods project, semi-structured interviews were conducted with 30 Swiss-based researchers involved in animal research and thematically analysed independently by two researchers. Preregistration was described as uncommon in animal research, with views varying by prior experience. Researchers with preregistration experience reported more positive views, whereas those without were more critical. Main barriers included administrative burden, time constraints, limited flexibility and perceived incompatibility with exploratory research. Reported benefits comprised improved study planning, adherence to good research practices and increased transparency. Suggestions to improve uptake involved training, institutional support, integration with the existing authorization procedure and maintaining voluntary participation. Preregistration in animal research remains uncommon and is shaped by prior experience. Addressing barriers through training, institutional support and structural integration may broaden its adoption.

## 1. Introduction

There has been a noticeable transition in recent years to open science practices within the scientific community, with the intention to strengthen transparency and scientific rigour, and to improve the replicability and reproducibility of research. This shift is partly driven by the so-called ‘replication crisis’ [1], which has shed light on sources of poor reproducibility of research findings, spanning from the humanities [2,3] to biomedical research [4]. The consequences of irreproducible research outcomes are far-reaching, as it undermines scientific credibility [5], raises ethical concerns [6] and erodes public trust in science [7]. Low rates of translation from preclinical to clinical research and poor reproducibility in animal research [1,8–11] have also raised concerns about irresponsible use of animals in research and putting patients in clinical trials at risk [12–15]. Although it is widely recognized that rigour in the design, conduct and reporting of research needs to improve, adoption of open science practices remains uneven across fields of research, with social sciences leading the way [16,17] and biomedical research slower in uptake [12].

One particularly promising open science practice is preregistration [18]. Defined as the registration of a study's hypotheses, methods and planned analyses in open repositories prior to data collection or data analysis [19–22], preregistration has been reported to help reduce questionable research practices, such as *p*-hacking, selective reporting and HARKing (‘hypothesizing after the results are known’) [21,23–26], as well as reducing publication bias and facilitating the distinction between planned and unplanned (exploratory) analyses, thereby improving scientific rigour [18,20,27,28]. There is growing empirical evidence indicating that preregistered studies report more null results and smaller effect sizes than non-preregistered studies, consistent with selective reporting and publication bias [29–34]. Together with larger sample sizes, better reporting and higher methodological quality [26,29,32,35], these findings highlight the potential of preregistration in promoting scientific rigour, transparency and reproducibility in animal research [36].

Despite the potential benefits of preregistration in mitigating questionable research practices and the availability of two animal research registries [19,22], its adoption remains low in Switzerland [37] and internationally [12]. Efforts to promote preregistration have emerged, with some journals encouraging preregistration through their publications policies [12]. In addition, a report by a US National Institutes of Health working group recommended preregistration for animal studies [38], and a recent call has advocated for its implementation in preclinical research [39]. However, broader uptake may depend on addressing perceived barriers, yet evidence on factors facilitating preregistration remains limited. A better understanding of animal researchers’ perspectives may inform strategies to support its adoption. This study therefore aimed to:

— Obtain in-depth insights into animal researchers’ experiences with preregistration.
— Explore animal researchers’ views on preregistration.
— Identify perceived barriers and facilitators to preregistration.
— Gather suggestions to improve the preregistration process and increase its adoption.

By addressing these aims, the present work seeks to generate a comprehensive understanding of factors influencing preregistration in animal research, offering insights into its applicability and informing future policy and infrastructure development.

## 2. Methods

This qualitative study is part of a larger cross-sectional mixed-methods project including two additional components: a quantitative study [37] and a systematic literature review [40]. The current study was preregistered on the Open Science Framework platform on 20 August 2024 (https://doi.org/10.17605/OSF.IO/KGV6R). A minimal deviation from the preregistered recruitment plan was made and is listed in table S1 in the supplementary material. The deviation is described following the guideline of Willroth & Atherton [41], and the study is reported in accordance with the Standards for Reporting Qualitative Research (SRQR) in table S2 in the supplementary material [42].

### 2.1. Qualitative approach and research paradigm

The study was based on a qualitative design, and thematic analysis guided data interpretation. This flexible analytical framework is often used to identify recurring patterns in qualitative data through systematic coding of the interviews, leading to the organization and identification of key themes derived from the interpretation of participants' narratives [43–45]. Furthermore, the study was framed within an interpretivist research paradigm, which assumes that multiple socially constructed realities coexist and enables researchers to develop a contextualized interpretation of the studied phenomena [46,47]. This paradigm was selected to explore participants’ lived experiences and perceptions of preregistration, emphasizing their subjective understanding rather than the objective measurement of these outcomes.

### 2.2. Researcher characteristics and reflexivity

All interviews were conducted in English by a female postdoctoral researcher (C.P.) with training and experience in qualitative data collection. The interviewer had a background in psychology and experience with preregistration practices, but did not have expertise in animal experimentation in Switzerland. To ensure an accurate understanding of the context, she familiarized herself with the Swiss legal framework and procedures for animal research and held regular meetings with the study supervisor (H.W.) to minimize bias and avoid potential misconceptions. Reflexivity was addressed through ongoing discussions within the research team that questioned assumptions and refined interpretations. The interviewer had no prior relationship or contact with participants before the interviews, except for three researchers she had briefly met in an academic context.

### 2.3. Context

This study was conducted in Switzerland, where animal research is regulated by the Animal Welfare Act, the Animal Welfare Ordinance and the Animal Experimentation Ordinance. Swiss legislation is among the most stringent in the world, as all animal experiments undergo a mandatory authorization process, overseen by the competent cantonal authority and informed by an ethical review by the cantonal advisory committee on animal experimentation. This procedure aims to guarantee compliance with good research practice, implementation of the 3R principles (replacement, reduction and refinement) and proportionality based on a formal harm–benefit analysis [48,49]. Applications are submitted confidentially via the federal platform animex-ch and are not publicly available. Study directors are legally responsible for ensuring that animal experiments are conducted in accordance with national and cantonal legislation. Despite being a formal legal designation, the role of study director corresponds to that of a principal investigator. This population was sampled because study directors hold decision-making authority regarding whether to preregister a study. To be eligible as a study director, researchers need a biomedical university degree, a minimum of 3 years of experience in animal research and the completion of a certified further education course in laboratory animal science. Participants in this study were drawn from the population of licensed study directors in Switzerland.

### 2.4. Sample strategy

#### 2.4.1. Targeted study sample

Although previous research suggests that thematic saturation is often achieved with around 15–20 interviews [50], a slightly higher number of 32 participants was targeted to capture diversity in preregistration experience, gender, language and seniority level (supplementary material, figure S3). This targeted sample was designed using a hierarchical stratification approach, aiming to include two equally sized subgroups of 16 researchers each—with and without prior preregistration experience. Distinguishing between researchers with and without preregistration experience was considered particularly relevant, as prior experience has been shown to be associated with a range of perceptions and experiences related to preregistration, including attitudes, perceived barriers and engagement with the practice [37]. This larger number of participants also allowed for a rich and diverse dataset across the two subgroups. Thus, each subgroup was structured to ensure balanced representation of gender (eight females and eight males), language region (four German-speaking and four French-speaking within each gender) and seniority (two early-career and two senior researchers within each language group). These characteristics were included to capture potential variation in perspectives across sociocultural contexts, rather than being based on an *a priori* expectation of differences in preregistration uptake as a function of these variables. In particular, stratification by language region aimed to ensure representation of researchers from the different linguistic regions of Switzerland, given the country's multilingual context. Language region was classified based on the location of the participants’ institutional affiliation, while seniority was determined by a combination of research experience, leadership responsibilities and institutional status. Participants holding a professorship or group leadership role were categorized as senior researchers, whereas all others were classified as early career researchers.

#### 2.4.2. Recruitment strategy

As this study was embedded in a larger mixed-methods project, participants for the interviews were recruited via an item in the online questionnaire used in the quantitative component, administered between May and June 2024 [37]. Interested individuals, or those referring other animal researchers, were redirected from the survey to a separate page to provide contact information. Their details were stored independently of the survey responses to ensure confidentiality. Potential participants were then classified according to their preregistration experience, gender, language and seniority level before being randomly selected and invited to participate in an interview. Given the limited availability of participants with prior preregistration experience, snowball and purposive sampling were additionally employed. First, interview participants were invited to name potential participants with preregistration experience. Second, two registries for preregistering animal studies—*animalstudyregistry.org* and *preclinicaltrials.eu*—were searched to identify researchers affiliated with Swiss institutions. Finally, a follow-up invitation email was sent to participants who had completed the online questionnaire. The message invited researchers with prior preregistration experience to take part in the qualitative study and to share their contact information via a link included in the email.

#### 2.4.3. Final study sample

For the subgroup of researchers without preregistration experience, recruitment proceeded as planned, and the targeted hierarchical stratification was achieved without difficulties. By contrast, for the subgroup with preregistration experience, the final sample did not fully match the targeted stratification. As shown by the quantitative component of the mixed-methods project [37], only 10% (*n* = 42) of animal researchers in Switzerland had prior preregistration experience. Given that the population of animal researchers with preregistration experience was very small, it was not feasible to recruit participants who met all predefined strata. Consequently, we encountered considerable difficulty in meeting the stratification requirements envisioned for the subgroup with preregistration experience. While the differences in gender distribution and seniority were minor, larger discrepancies occurred in language region, with almost no representation of preregistration-experienced researchers from the French-speaking part of Switzerland. These differences reflect feasibility constraints in the available population rather than changes to, or deviations from, the preregistered sampling plan. Nevertheless, the missing strata representation was considered acceptable and the resulting sample (supplementary material, figure S4) was adequate to support a rich and diverse dataset.

### 2.5. Ethical issues pertaining to human subjects

The study received ethical approval from the Ethical Commission of the Faculty of Psychology at the University of Bern, Switzerland (no. 2024-03-06). All participants were informed about the scope of the study, the parties involved and the funding source. Written informed consent was signed by all included participants and blank copies of the informed consent forms used for the in-person and online interviews are provided in consent forms S5 and S6 in the supplementary material, respectively. For the in-person interviews, participants signed the informed consent prior to the interview. For the online interviews, verbal consent was received before the recording began, and participants were instructed to email the signed consent form to the study team following the interview. Data were stored on a secured server at the University of Bern, accessible only to authorized study personnel. The interview recordings were deleted after the publication of the current article.

### 2.6. Data collection methods

Participants were contacted by email to arrange interview appointments. Individual semi-structured interviews were conducted either in-person or via Zoom, according to participants’ preferences. Pilot interviews took place between 08 May 2024 and 03 July 2024, while study interviews were held between 04 September 2024 and 10 January 2025. To ensure a consistent understanding of the topic, the definition of preregistration was provided before the interview began (supplementary material, definition S7). All interviews were audio-recorded and manually transcribed verbatim by one researcher (S.M.L.), while a second researcher (C.P.) reviewed the transcripts for accuracy. Personal information was anonymized during transcription to protect participants’ confidentiality.

### 2.7. Data collection instruments and technologies

A semi-structured interview guide was developed based on the study objectives and the available literature. The guide was revised through several rounds by the study team and pilot-tested with three interviewees. These steps allowed the refinement of the open-ended questions and improved the flow and clarity of the interview structure. Two finalized versions of the guide were used with the study sample, depending on participants’ prior experience with preregistration ( table 1). In addition, a brief questionnaire was administered to collect sociodemographic information, including age, gender, years registered as study director, years of animal research experience, academic age, educational attainment, seniority level, field of animal research and organizational affiliation.

**Table 1.** Semi-structured interview guide.

Background information
**1. To start with, could you tell me a little bit about your research topic and what you are currently working on? This way, I can get a better understanding of the work you conduct.**
a) What types of animal studies do you typically conduct?
b) How long have you been working in this field
**Experience with preregistration**
**2. Moving on now to the topic of this interview, I would like to ask you in which context did you first learn about preregistration?**
**3. What is your understanding of study preregistration? Can you describe what you think preregistration involves?**
**Facilitators and barriers**
**4. Have you ever preregistered a study?**
*if no*
a) What are the reasons for not preregistering your studies?
b) Can you describe any specific difficulties you would anticipate when preregistering a study?
c) What would make you consider preregistering your studies in the future?
*if yes*
a) What are the reasons for you to preregister your studies?
b) Can you describe the process of preregistration and your experience with it?
c) What specific difficulties have you encountered while preregistering your studies?
d) Have you experienced any benefits or positive outcomes from preregistering your studies?
**General views**
**5. What are your general views on the concept of preregistration for animal studies?**
a) What do you think are the advantages?
b) What do you think are the disadvantages?
**6. In your opinion, does preregistration impact the scientific quality of animal research? How exactly?**
**7. Do you personally find preregistration to be more beneficial or more problematic?**
a) Can you explain why?
**8. How do you think preregistration is perceived and used in your research field?**
**Suggestions for improvement**
**9. What do you think would make other animal researchers preregister their studies?**
**10. What kinds of support or resources would help you and your colleagues better engage with study preregistration?**
**11. What suggestions do you have for improving the process of study preregistration in animal research? Are there specific changes you would recommend?**
a) What role should institutions, funding bodies, publishers or regulatory agencies play in facilitating preregistration?
**12. What are your thoughts on making study preregistration mandatory in animal research?**
**13. Looking ahead, what do you think the future of study preregistration in animal research will look like?**
a) In your opinion, what should preregistration look like?
b) What long-term benefits or challenges do you anticipate?
**Closing**
**14. We are now at the end of the interview, and I want to thank you for your participation and your valuable input. Is there anything else you would like to add regarding the preregistration of animal studies that has not yet been covered? Are there any final thoughts you would like to share?**

### 2.8. Units of study

The study units consisted of individual animal researchers who served as study directors and held an active licence for animal experimentation either at the time of data collection or in the past, as well as individuals with professional experience in animal research who did not hold such a licence.

### 2.9. Data processing

The qualitative data analysis software MAXQDA was used to assist with the thematic analysis of the interviews [51]. This software facilitates the management of qualitative datasets by supporting the systematic application of codes and allowing the exploration and analysis of coded material.

### 2.10. Data analysis

Following transcription, systematic coding of the data was conducted to identify recurring patterns and generate themes through an iterative and interpretative process. Codes were generated through careful and repeated reading of the transcripts, with segments of the transcribed interview text assigned labels capturing relevant features of the dataset. Coding was conducted using MAXQDA, which helped organize the transcripts, assign and manage codes and retrieve coded segments for comparison during theme development. The codes were subsequently examined, and those clustering around similar topics were developed into specific themes. Themes were further reviewed and refined by examining the coherence of coded data within each theme and the distinctions between themes.

This process was conducted collaboratively by the first author (C.P.) and a student research intern (G.S.). Both researchers independently coded the same transcripts and met regularly throughout the coding process to compare coded sections, discuss differences in coding and interpretation and review newly developed codes. The coding framework was iteratively refined based on these discussions as the analysis progressed. Differences were resolved through discussion. Because the purpose of these discussions was to refine the coding and interpretation collaboratively, rather than to quantify agreement between coders, no formal measure of intercoder agreement was calculated. Themes were therefore inductively developed through researchers’ interpretation of the data and represent patterns identified within the dataset. Thematic analysis continued until sufficient depth and richness of themes were achieved, suggesting a meaningful capture of the patterns in the dataset.

All codes and subcodes generated by both researchers informed the development and refinement of the final themes, although not all individual codes and subcodes are explicitly presented in the manuscript. Complete overviews of the coding systems used by C.P. and G.S. are provided in tables S8 and S9, respectively, in the supplementary material.

### 2.11. Techniques to enhance trustworthiness

Several strategies were implemented to ensure the trustworthiness of the analysis. First, purposive sampling aimed to capture a diverse range of perspectives across preregistration experience, gender, linguistic region and seniority in the study sample, enabling the exploration of variation within the dataset. Second, investigator triangulation [52] was achieved by involving three researchers—one with preregistration experience (C.P.) and two without (S.M.L. and G.S.)—who independently reviewed the transcripts, coded the data and collaboratively engaged in data interpretation and thematic development. This approach allowed for the integration of diverse perspectives into the analysis and enhanced its depth and reliability. Third, reflexivity was maintained throughout the study via regular team discussions within the research team, supporting transparency in the analytic process. Fourth, the study was preregistered prior to data collection, which enhanced transparency and methodological rigour. A single minimal deviation from the preregistered recruitment plan occurred (supplementary material, table S1) without affecting the analysis or the resulting interpretations. Finally, findings from this qualitative study were interpreted in conjunction with results from the quantitative component of the broader mixed-methods project, representing a form of data triangulation across datasets at the level of interpretation. Taken together, these techniques enhanced the overall credibility and robustness of the results.

## 3. Results

### 3.1. Interview description

A total of 34 interviews were conducted, which lasted between 17 and 52 minutes (*M* = 30.1 min, s.d. = 8.6). Four interviews took place in-person at locations chosen by the participants, while 30 were held online via Zoom. The three pilot interviews were not included in the final analysis. One additional interview was excluded from the analysis as the participant did not return the signed consent form following the online meeting. The final dataset for this qualitative study comprised 30 interviews.

### 3.2. Sample description

On average, participants were 45.3 (s.d. = 9.5) years old, and slightly more women (53.3%) than men (46.7%) agreed to participate in the study (table 2). The sample was highly educated, with all participants holding a PhD, Dr. med. or Dr. med. vet. title and nearly half (46.7%) having completed a habilitation or holding a professorship. Participants had substantial professional experience (years since registration as study director: *M* = 9.4, s.d. = 7.5; years of research experience: *M* = 20.8, s.d. = 9.8; academic age in years: *M* = 14.1, s.d. = 9.3), came mostly from basic and experimental medical research (78.6%), and the majority were employed in academic institutions (90%). There was a slight difference in preregistration experience within the sample, with fewer participants classified as having preregistration experience (*n* = 14) than having no prior experience (*n* = 16). Among those with preregistration experience, nine participants had preregistered at least one animal study in the classical sense, while the remaining participants had more varied preregistration backgrounds: one had not preregistered a study themselves but had supervised a postdoctoral researcher who had preregistered (ID 5006); one had experience with both preregistration and registered reports (ID 5016); one had begun preregistering a study but ultimately decided not to proceed (ID 5025); one had preregistered systematic reviews but not animal studies (ID 5029) and one described a voluntary protocol submitted abroad alongside the formal animal experimentation licence, which did not constitute preregistration in the classical sense but represented a related variation of it (ID 5013). Given the very small number of animal researchers in Switzerland with preregistration experience, all participants with any form of preregistration experience were included in this subgroup.

**Table 2.** Sample description.

characteristics	participants (*n* = 30)
**age**	
*M* (s.d.)	45.3 (9.5)
*Mdn*	43.0
range	31–64
*n*	30 (0 missing)
**gender**	
female	53.3% (16)
male	46.7% (14)
*n*	30 (0 missing)
**years registered as study director**	
*M* (s.d.)	9.4 (7.5)
*Mdn*	7.0
range	2–28
*n*	27 (3 missing)
**animal research experience**	
*M* (*s.d.*)	20.8 (9.8)
*Mdn*	18.5
range	7–44
*n*	30 (0 missing)
**academic age**	
*M* (s.d.)	14.1 (9.3)
*Mdn*	10.6
range	2–39
*n*	30 (0 missing)
**educational attainment**	
PhD/Dr. med.	53.3% (16)
habilitation^a^/professorship	46.7% (14)
*n*	30 (0 missing)
**seniority level**	
lecturer	3.4% (1)
postdoc./senior researcher	31.0% (9)
project leader/group leader	27.6% (8)
professor	31.0% (9)
other	6.9% (2)
*n*	29 (1 missing)
**field of animal research**	
basic and experimental medical research^b^	78.6% (22)
general biology^c^	17.9% (5)
basic biological research^d^	3.6% (1)
*n*	30 (2 missing)
**organizational affiliation**	
academic	90.0% (27)
private	6.7% (2)
academic & private	3.3% (1)
*n*	30 (0 missing)

Note: *M* = mean; s.d. = standard deviation; *Mdn* = median; *n* = total sample size.

^a^ Postdoctoral qualification required for independent professorship in German-speaking European countries.

^b^ Including following fields: biomedical engineering; cancer research; cardiovascular research; endocrinology; immunology; medical microbiology; neuroscience; nutrition and metabolism; pathology and/or pathophysiology; pharmacology; physiology; regenerative medicine; toxicology; veterinary medicine; virology.

^c^ Including following fields: animal breeding; animal nutrition; animal welfare; ecology; ethology; evolution; laboratory animal science; wildlife biology; zoology.

^d^ Including following fields: biochemistry; biophysics; cell biology; cytology; developmental ciology; embryology; epigenetics; experimental microbiology; genetics; molecular biology; radiobiology; structural biology.

### 3.3. Thematic analysis

Recurring patterns and themes were identified and organized in relation to the overarching aims of the study, namely participants’ experiences with preregistration, their general views, perceived barriers and facilitators and suggestions for improvement (see figure 1 for an overview of the main findings).

**Figure 1.**
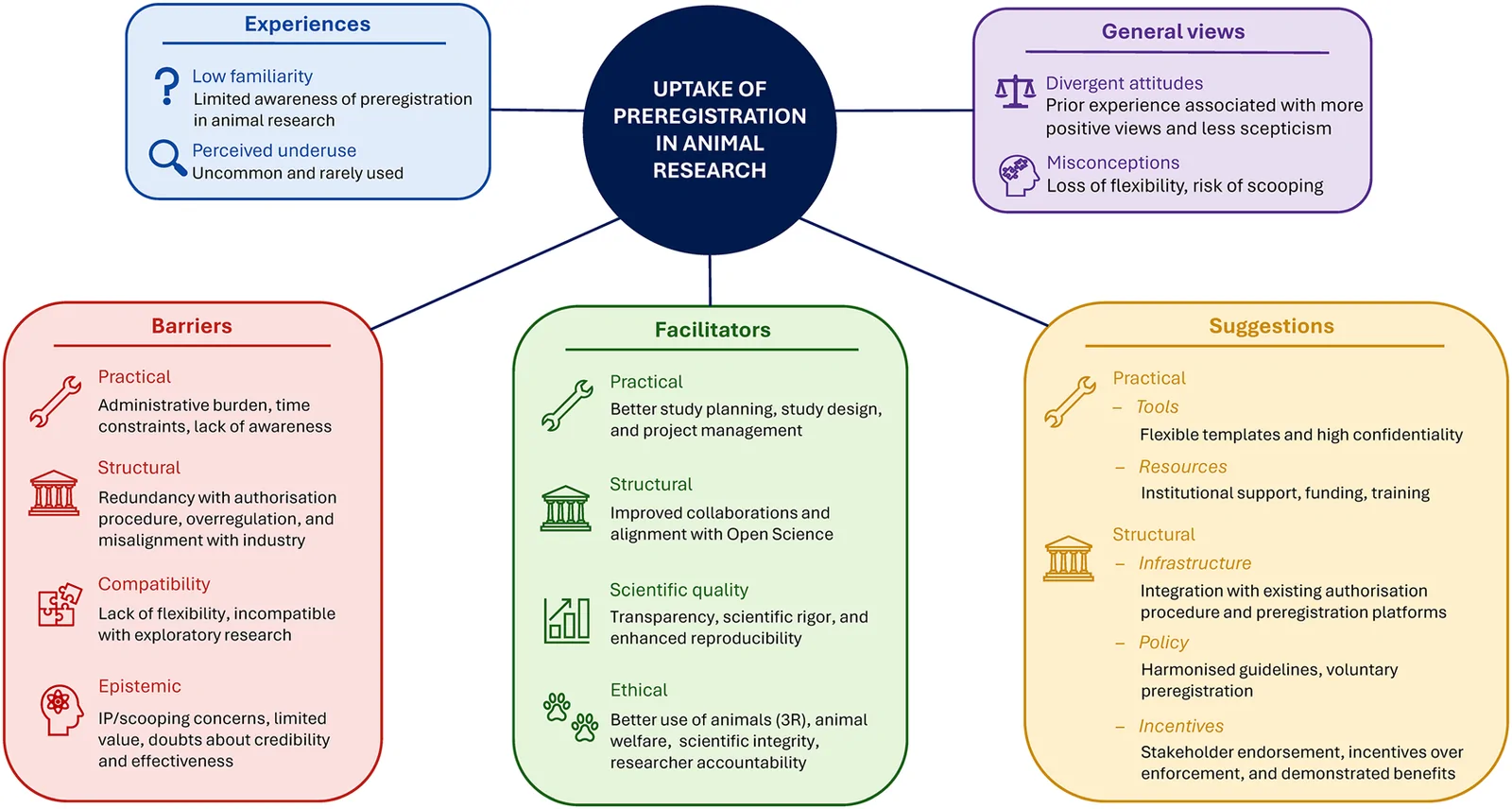
Thematic analysis.

#### 3.3.1. Experiences with preregistration (aim 1)

Thematic analysis led to the identification of two main themes regarding participants’ experiences with preregistration: *low familiarity* with the practice and *perceived underuse* in the research community (figure 2).

**Figure 2.**
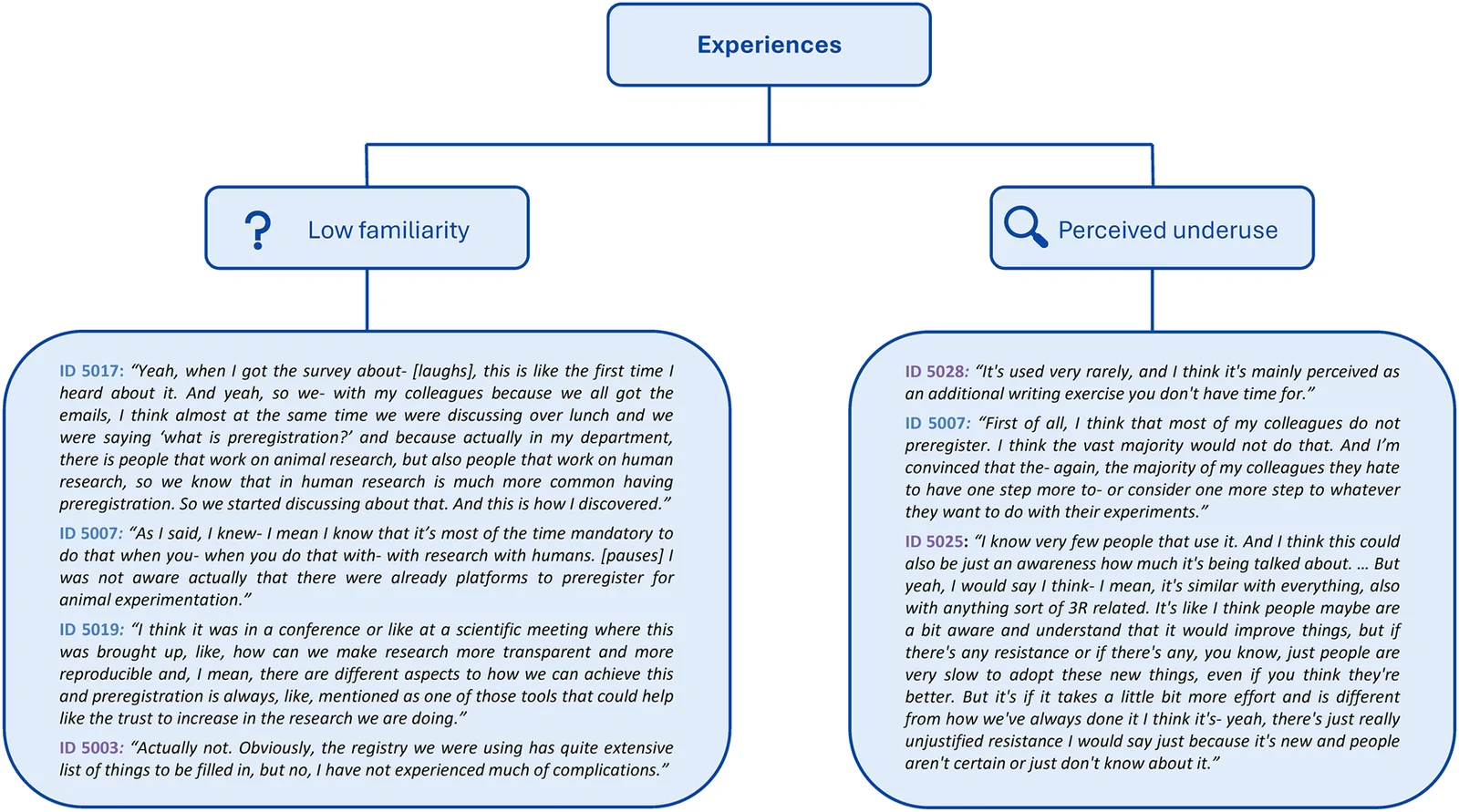
Experiences with preregistration. Participant IDs shown in purple indicate researchers with preregistration experience, while IDs shown in blue indicate those who had not preregistered any study prior to the interview.

##### Low familiarity

Several participants without prior preregistration experience had never heard of the practice before receiving our study invitation. Some, however, were aware of preregistration in the context of human clinical trials (figure 2: ID 5017) but had no knowledge of its use in animal research or of existing specialized platforms (figure 2: ID 5007). Participants with preregistration experience, in turn, were often introduced to it through colleagues, further educations courses or scientific meetings (figure 2: ID 5019), and had not encountered any problems with the preregistration process (figure 2: ID 5003).

##### Perceived underuse

Regardless of their own preregistration experience, participants generally perceived the practice as underused within their research field and noted a lack of willingness among researchers to engage in it (figure 2: ID 5028; ID 5007; ID 5025).

#### 3.3.2. General views (aim 2)

Two overarching themes capturing participants’ perceptions and views of preregistration were developed through thematic analysis: *divergent attitudes* towards preregistration and *misconceptions* associated with it (figure 3).

**Figure 3.**
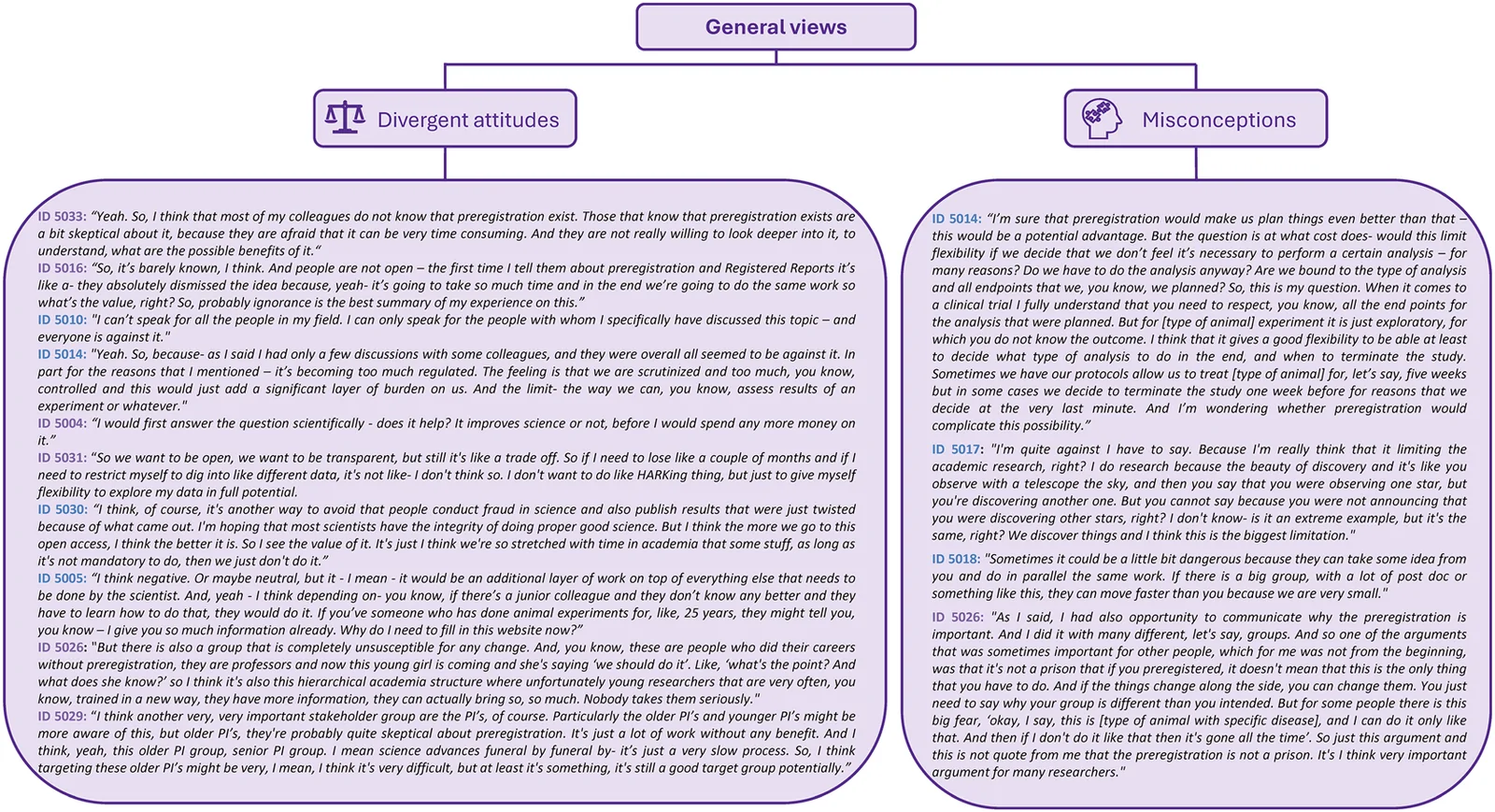
General view of preregistration. Participant IDs shown in purple indicate researchers with preregistration experience, while IDs shown in blue indicate those who had not preregistered any study prior to the interview.

##### Divergent attitudes

Participants described contrasting views of preregistration depending on their familiarity with the practice. Among researchers with prior preregistration experience, views were predominantly positive, with almost all expressing supportive attitudes and endorsing its broader use (figure 3: ID 5033; ID 5016). By contrast, researchers who had never preregistered a study generally expressed scepticism and more negative attitudes (figure 3: ID 5010; ID 5014). Within the preregistration subgroup, a few nuances were observed: one participant expressed a more reserved view towards preregistration and asked for more research and proof of its effectiveness (figure 3: ID 5004), while another described preregistration positively in general but was unsure how well it would align with their own research context (figure 3: ID 5031). Within the subgroup without preregistration experience, attitudes also varied, with a small subset expressing openness towards the practice and acknowledging its potential value (figure 3: ID 5030). Furthermore, regardless of their own preregistration experience, some participants anticipated that more senior researchers would likely oppose preregistration, arguing that their expertise and judgment should outweigh the need for formal registration (figure 3: ID 5005; ID 5026; ID 5029). Taken together, attitudes towards preregistration were divergent across the sample, with predominantly positive views concentrated among participants with preregistration experience and more sceptical views among those unfamiliar with it.

##### Misconceptions

Most participants correctly described what preregistration entails, with only a few providing incomplete or partly inaccurate definitions. However, certain misconceptions were noted among some participants without preregistration experience. Several mentioned concerns that preregistration would limit flexibility during a study, fearing that necessary changes would not be possible and that they would be forced to adhere to a protocol that no longer worked or would not allow them to pursue alternative approaches not specified in the preregistration (figure 3: ID 5014; ID 5017). Others expressed concerns that competitors might scoop their research ideas, particularly larger or better-funded groups that could move more quickly and complete a study before them (figure 3: ID 5018). Those with preregistration experience, however, did not have these concerns and offered contrasting perspectives (figure 3: ID 5026).

#### 3.3.3. Barriers (aim 3)

Numerous barriers to preregistration were reported, particularly by participants who had never engaged in this practice. Thematic analysis identified and grouped these barriers into four main categories: *practical*, *structural*, *compatibility* and *epistemic* (figure 4).

**Figure 4.**
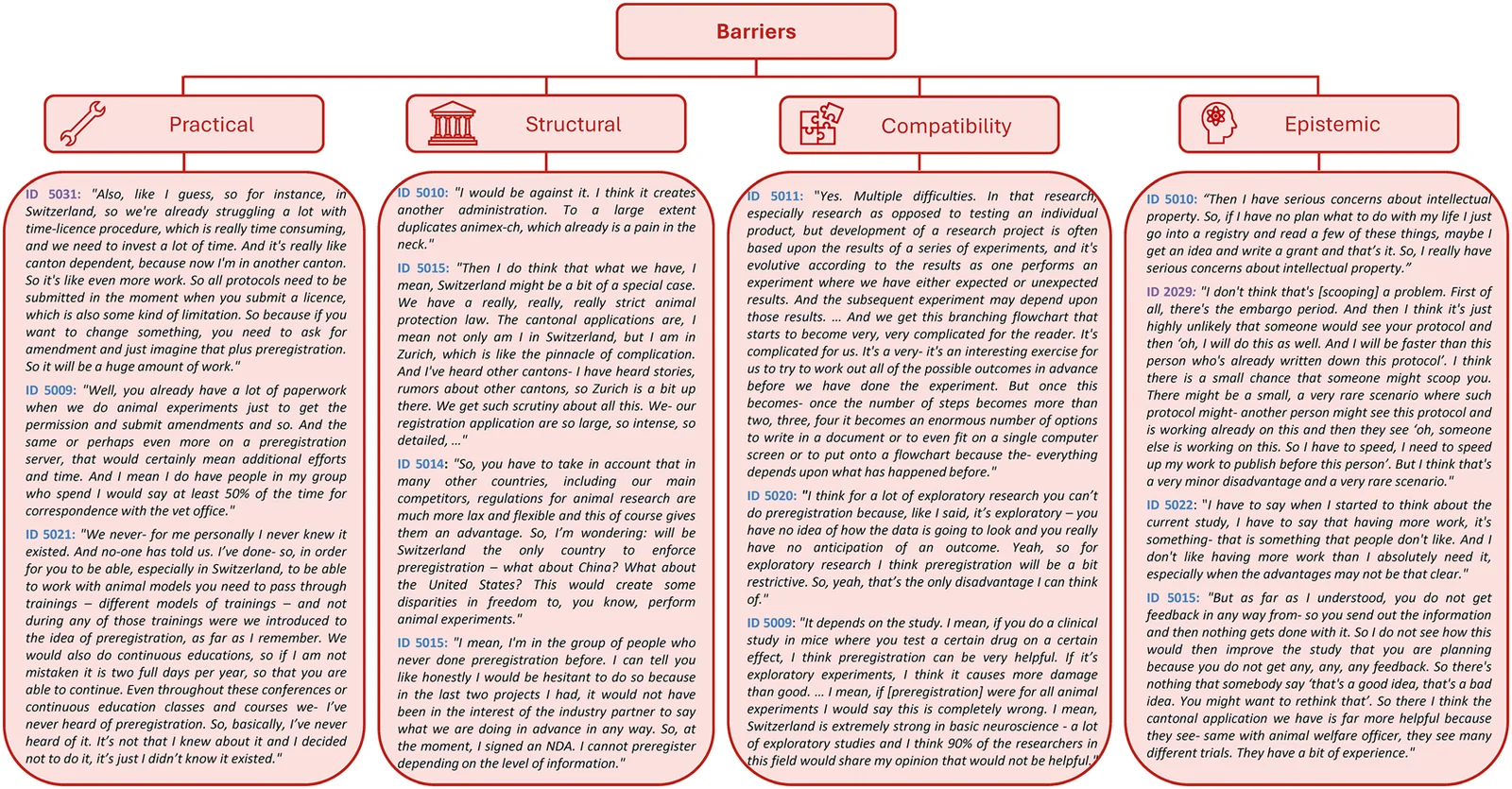
Barriers to preregistration. Participant IDs shown in purple indicate researchers with preregistration experience, while IDs shown in blue indicate those who had not preregistered any study prior to the interview.

##### Practical barriers

Practical barriers were mainly linked to workload, time investment and available resources. Regardless of their preregistration experience, the majority of participants complained about the heavy administrative burden associated with conducting animal research in Switzerland (figure 4: ID 5031). Many, especially those who had never preregistered, perceived preregistration as an additional bureaucratic step that requires considerable time to complete (figure 4: ID 5009). Furthermore, for several participants, a major barrier to preregistration was their lack of knowledge that such a practice existed (figure 4: ID 5021).

##### Structural barriers

Described structural barriers concerned the regulatory requirements for animal experiments in Switzerland, particularly the authorization procedure. A central issue, mostly raised by participants without preregistration experience, was the perceived redundancy of preregistration in relation to the authorization procedure. This was often attributed to animex-ch—a governmental web application used to record mandatory licence applications for animal experimentation (figure 4: ID 5010). Participants also criticized the overregulation of animal research in Switzerland, suggesting that further requirements could reduce the number of studies and weaken international competitiveness (figure 4: ID 5015; ID 5014). The perceived misalignment between preregistration and industry was also mentioned, as scientists in this sector either conduct internal preregistrations within their companies or refrain from preregistering altogether owing to confidentiality agreements (figure 4: ID 5015).

##### Compatibility barriers

Compatibility barriers were primarily concerned with the perceived feasibility of preregistration for certain research designs, as well as its flexibility and adaptability. As previously noted, several participants without preregistration experience raised concerns about the lack of flexibility that preregistration imposes on their methodologies. Many emphasized that preregistration may be unsuitable for exploratory or basic research owing to the unpredictable and often iterative nature of such studies, which often leads to deviations from initial plans, making preregistration, in their view, unfeasible (figure 4: ID 5011; ID 5020). Nevertheless, some participants saw potential for this practice in confirmatory research (figure 4: ID 5009).

##### Epistemic barriers

Participants reported several epistemic barriers to preregistration related to its credibility and perceived value. Concerns about intellectual property and idea scooping were the most frequently mentioned among researchers who had never preregistered (figure 4: ID 5010), with a few also expressing worries about potential harassment by activists. By contrast, those familiar with preregistration held opposite views on these issues (figure 4: ID 2029). Certain researchers without preregistration experience also expressed little interest in engaging with the practice or regarded it as unnecessary, viewing it as a measure that could restrict innovation. In addition, some participants emphasized that preregistration is not yet a commonly established practice within the animal research community, while others reported limited awareness of the potential benefits and impact (figure 4: ID 5022). Finally, a small group questioned its value, noting that preregistration records are not checked and no official feedback is provided (figure 4: ID 5015), with a few even suggesting that the system could be manipulated, as preregistration forms might be falsified.

#### 3.3.4. Facilitators (aim 3)

Facilitators to preregistering studies encompassed a range of perceived benefits associated with this practice, which clustered into four main themes: *practical*, *structural*, *scientific quality* and *ethical facilitators* (figure 5). These benefits were reported primarily by participants with prior preregistration experience, although some researchers unfamiliar with the practice also recognized its potential value. Nevertheless, a small subset without preregistration experience saw no benefit in preregistering animal studies and expressed strong opposition to its use.

**Figure 5.**
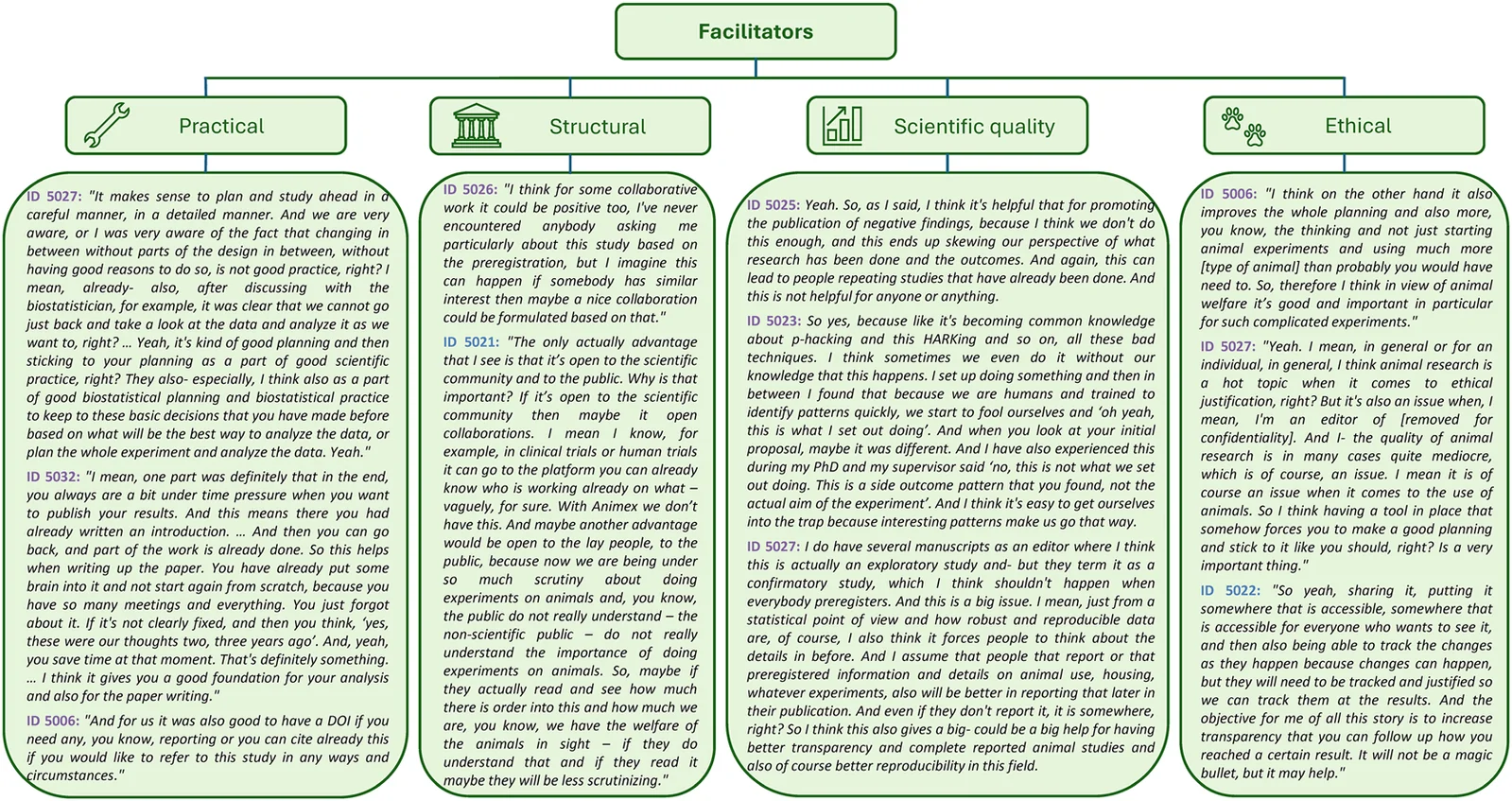
Facilitators to preregistration. Participant IDs shown in purple indicate researchers with preregistration experience, while IDs shown in blue indicate those who had not preregistered any study prior to the interview.

##### Practical facilitators

Several participants described practical facilitators related to workflow, noting that preregistration can support better study planning, improve time and project management and foster more thorough methods and stronger study designs (figure 5: ID 5027). In addition, preregistration was perceived to help with drafting papers and accelerating the publication process (figure 5: ID 5032). In contrast to participants without preregistration experience, who expressed strong concerns about scooping, those familiar with the practice described preregistration as protecting intellectual property by documenting their research steps (figure 5: ID 5006).

##### Structural facilitators

Structural facilitators referred to systemic and community-level factors that promote preregistration. Some participants mentioned that preregistration could facilitate collaborations and synergies, as well as encourage feedback and scientific exchange (figure 5: ID 5026). Others highlighted its alignment with Open Science initiatives and its role in fostering greater public trust in animal research (figure 5: ID 5021).

##### Scientific quality facilitators

Many participants discussed facilitators related to scientific quality, particularly concerning transparency, rigour and reproducibility. Preregistration was described as a tool to increase transparency in science, prevent publication bias and reduce parallel research (figure 5: ID 5025). Several participants emphasized its potential to prevent questionable research practices, such as HARKing, selective reporting and *p*-hacking (figure 5: ID 5023). Furthermore, some mentioned that preregistration could reduce deviations from protocols and minimize researcher bias. Those in favour of the practice believed that preregistration could enhance reproducibility in science and improve overall research quality (figure 5: ID 5027).

##### Ethical facilitators

Ethical facilitators reflected aspects related to animal welfare and scientific integrity. Certain participants reported that preregistration promotes better consideration of animal use in line with the 3Rs (Replacement, Reduction and Refinement) and can therefore enhance animal welfare (figure 5: ID 5006; ID 5027). Others noted that preregistration fosters greater accountability by transparently documenting all study steps and reinforcing researchers’ responsibility in animal experimentation (figure 5: ID 5022).

#### 3.3.5. Suggestions for improvement (aim 4)

Participants proposed a wide range of suggestions to improve the preregistration process and increase its uptake. No systematic differences were observed between participants with and without preregistration experience in the types of suggestions they proposed. Following thematic analysis, these recommendations were organized into *practical* and *structural suggestions* (figure 6).

**Figure 6.**
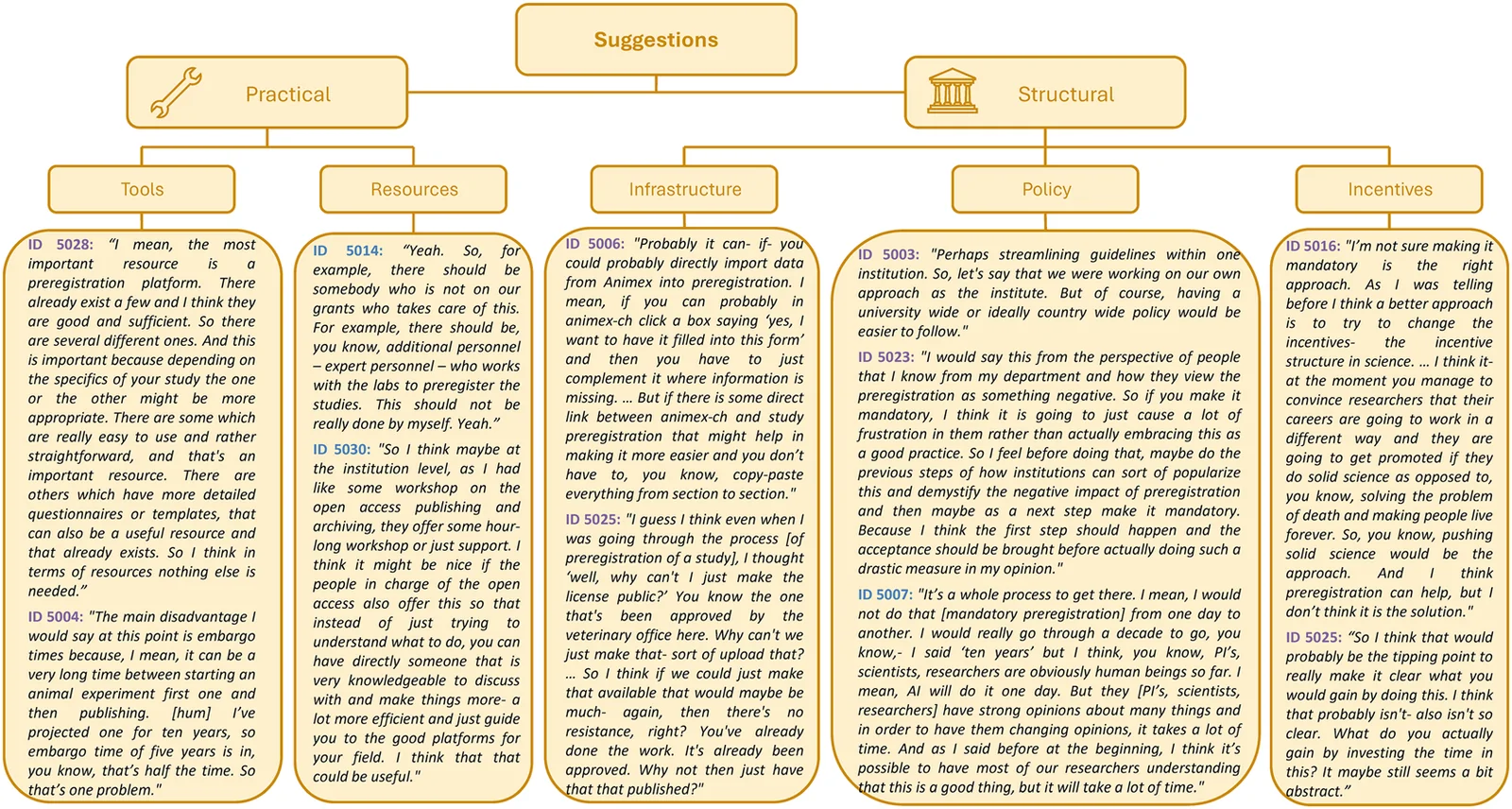
Suggestions for improvement. Participant IDs shown in purple indicate researchers with preregistration experience, while IDs shown in blue indicate those who had not preregistered any study prior to the interview.

##### Practical suggestions

Practical suggestions addressed aspects related to templates and support required to facilitate preregistration and were further divided into two subthemes: tools and resources (figure 6).

— Tools: participants emphasized the development of easy-to-use and flexible templates and suggested exploring AI-based tools to assist with preparing preregistrations (figure 6: ID 5028). Some participants also highlighted the importance of strong confidentiality measures and longer embargo options (figure 6: ID 5004).
— Resources: beyond technical support, participants called for additional resources and institutional help, such as dedicated staff, funding and assistance from animal welfare offices (figure 6: ID 5014). They further recommended organizing workshops and training courses to increase researchers’ awareness and familiarity with preregistration and providing clear guidance on where and how to preregister (figure 6: ID 5030).

##### Structural suggestions

Structural suggestions focused on necessary changes to the current authorization procedure and the existing institutional policies, and were further grouped into three subthemes: infrastructure, policy and incentives (figure 6).

— Infrastructure: participants proposed several approaches to integrate preregistration into existing systems. One suggestion was to link preregistration with licence applications for animal experiments by incorporating it into animex-ch, the governmental platform used to submit animal experiment authorization applications, to avoid duplication (figure 6: ID 5006). Others recommended connecting animex-ch to an existing international preregistration platform rather than creating a new Swiss registry. Additional proposals included making animex-ch applications publicly available (figure 6: ID 5025) or enabling internal preregistration processes through university ethics committees.
— Policy: proposed policy-related suggestions included harmonizing guidelines across institutions and promoting the international implementation of preregistration beyond Switzerland (figure 6: ID 5003). Furthermore, most participants favoured keeping preregistration voluntary, noting that mandatory implementation could prompt strong negative reactions within the research community (figure 6: ID 5023). Some, however, supported a gradual implementation of mandatory preregistration or restricting it to confirmatory research (figure 6: ID 5007).
— Incentives: participants emphasized the importance of endorsement from key stakeholders, including institutions, funders, journals, regulatory bodies and the scientific community. Incentives such as publishing benefits, funding advantages, career rewards and streamlined authorization processes for preregistered studies were generally preferred over enforcement measures (figure 6: ID 5016). Many participants also stressed that clearly demonstrating the benefits of preregistration would be essential to encourage broader engagement within the research community (figure 6: ID 5025).

## 4. Discussion

The present study constitutes the qualitative component of a comprehensive mixed-methods project on the current practices and attitudes of animal researchers concerning preregistration of animal experiments in Switzerland. To our knowledge, this is one of the first studies to qualitatively assess animal researchers’ experiences and views regarding preregistration, identify potential barriers and facilitators and summarize suggestions for improving its implementation and uptake.

### 4.1. Experiences with preregistration

The interviews revealed low familiarity with preregistration both among participants and within their research communities. This finding was consistent with results from the survey conducted as part of the quantitative component of the mixed-methods project to which the current study belongs. In the survey, only 10% of respondents had ever preregistered a study, and 39% had never heard of preregistration before participating [37]. Together, these results provide methodological triangulation, confirming low awareness of preregistration in the field, across both qualitative and quantitative datasets. Our results stand in contrast to evidence from psychology, experimental economics and the social and behavioural sciences, where preregistration is better known and more commonly practiced [17,27,53–56]. This indicates that preregistration is at an early stage of adoption in animal research, highlighting the need to promote awareness and facilitate uptake in this field. In addition, participants without preregistration experience in the current study varied in their prior knowledge of the practice. Similar to the related survey, 6 of the 16 participants (37.5%) had never heard about preregistration before, while others had some prior awareness. Limited familiarity may have contributed to more sceptical attitudes and perception of preregistration as challenging or burdensome.

### 4.2. General views

Consistent with the potential role of familiarity in shaping perceptions of preregistration, we observed divergent views on preregistration depending on participants’ prior experience with it. Those with preregistration experience generally expressed more positive views, whereas those without such experience tended to be more critical. This pattern was also seen in the complementary survey study, where preregistration experience emerged as the strongest predictor across all analysed constructs, being associated with more positive attitudes, norms, intentions and motivations, as well as fewer perceived obstacles to preregistration [37]. Furthermore, our qualitative analysis revealed that senior researchers are more resistant to preregistration. This pattern was also mirrored in a qualitative study of German stakeholders of animal research [36], as well as in our quantitative survey study [37] and studies from behavioural science and psychology [27,53]. The agreement between the qualitative findings presented here and results from our complementary quantitative data, together with findings from other disciplines, provides cross-validation of our results and suggests that familiarity and exposure to preregistration, as well as researchers’ career stage, may play important roles in shaping researchers’ perception of the practice. Given the observational nature of these studies, however, no conclusions can be drawn regarding the direction of these associations. Future efforts to promote preregistration among animal scientists should take the differing perspectives and concerns identified across these specific subgroups into account. For example, including preregistration in undergraduate teaching of methods and giving early career researchers exposure to preregistration (e.g. in dedicated courses or workshops) might be particularly sustainable interventions.

### 4.3. Barriers

Our analysis revealed contrasting perceptions among participants regarding the barriers and facilitators to preregistration. With few exceptions, those who had never engaged in preregistration reported numerous barriers, whereas participants with prior experience emphasized a broad range of benefits associated with it. The main barriers concerned the substantial bureaucratic burden already associated with conducting animal research under Switzerland's regulatory framework and the belief that preregistration would be too time-consuming, further increasing researchers' administrative workload. These two barriers were also the most frequently reported by participants in our quantitative study [37], and were identified as weaknesses of preregistration in both a qualitative and a quantitative study conducted in Germany among animal researchers [36,57]. These findings are also consistent with results reported in psychology and reflect broader cross-disciplinary and cross-method perceptions of preregistration as time-consuming [53]. In addition, fears of idea scooping and concerns about losing flexibility in conducting research were further barriers highlighted by participants unfamiliar with preregistration. These assumptions reflect misconceptions of the preregistration process. Study plans can be modified as needed through amendments, and deviations can be transparently reported in publications, while preregistration records can be embargoed until the corresponding papers are published. Consistent with these findings, our quantitative study showed that fears of scooping ranked among the top five concerns for researchers who had never preregistered, but did not appear among the top five actual problems encountered by those with preregistration experience [37]. Potential theft of ideas was also reported as a perceived weakness by animal scientists in the two German studies, but it was rated as minor and preregistration was described as a means of protecting intellectual property from scooping [36,57]. In line with these concerns, another major barrier revealed by our study was the perceived incompatibility of preregistration with exploratory and basic research. Researchers whose work involves greater methodological flexibility appeared more sceptical of preregistration than those conducting studies in which procedures, tests and methods can be defined *a priori*. Nevertheless, participants acknowledged the potential of preregistration for hypothesis testing and other forms of confirmatory research. Taken together, our findings indicate that perceived barriers to preregistration stem from practical challenges and conceptual misunderstandings of the process, as well as structural and methodological constraints specific to animal research. Addressing these misconceptions through education and reducing administrative hurdles (as further discussed in §4.5) may therefore be essential to improving acceptance and uptake of preregistration within the animal research community.

### 4.4. Facilitators

Apart from barriers, our thematic analysis identified several facilitators of preregistration, primarily reported by participants with prior experience of using it. Preregistration was perceived as a tool offering practical and structural benefits, including improved study planning, stronger study methodology and more efficient project management, as well as fostering collaboration, alignment with Open Science initiatives and greater public trust in animal research. In addition, preregistration was associated with scientific and ethical advantages, such as increased transparency, reduced questionable research practices and greater accountability, leading to better adherence to the 3Rs principles and more responsible animal use. These findings are consistent with the German stakeholder analysis of animal study registries, which identified similar benefits, including improvements in study planning, transparency and research quality, as well as potential benefits for collaboration, resource use and public trust [36]. Collectively, these findings suggest that preregistration is viewed as having the potential to enhance research quality and integrity, as well as to strengthen ethical standards and external trust in animal research.

### 4.5. Suggestions for improvement

Several suggestions for improving the practice and uptake of preregistration were proposed by participants with both favourable and critical views of the practice. Participants emphasized the need to raise awareness and to provide training on the process and practical steps of preregistration, along with efforts to demonstrate its scientific benefits. Recommendations for simplifying procedures and linking preregistration to the authorization procedure for animal experiments were also made, with the aim of reducing administrative burden and streamlining the process. For example, by adjusting the template of the application form for the authorization of animal experiments to the needs of a preregistration such that the same form would serve both purposes, seems particularly promising in this respect. Importantly, the majority of participants stressed that preregistration should remain voluntary, with a focus on introducing incentives rather than enforcing mandatory participation. They noted that the research community may otherwise respond negatively, as many scientists are not yet sufficiently informed or convinced about the practice and would therefore require additional guidance and support. As mentioned by some participants, funder policies recommending preregistration could be a promising incentive, such as the policy by the Dutch organization for knowledge and innovation in health, healthcare and well-being (ZonMw; https://www.zonmw.nl/en/animal-wise), or The German Centre for the Protection of Laboratory Animals (Bf3R; https://www.bf3r.de/en/offers/foerderung/bf3r-research-funding/).

### 4.6. Strengths and limitations

The central strength of the current study lies in its qualitative design, which enabled an in-depth exploration of researchers’ perspectives and provided insight into how preregistration is experienced and understood in practice. Furthermore, the study was embedded within a larger mixed-methods project and complemented by a quantitative survey, allowing for methodological triangulation that cross-validated the findings and strengthened the overall conclusions. Nonetheless, given the qualitative design used, several limitations should be considered. First, the sample may have been subject to selection bias, as participation was voluntary, which may have attracted researchers with particularly strong opinions (both positive and negative) on preregistration. Nevertheless, extensive efforts were made to achieve hierarchical stratification by including animal researchers who differed in preregistration experience, gender, linguistic region and seniority. This diverse sample enabled us to capture a wide range of perspectives and experiences, identify unique challenges faced by subgroups within our participant pool and enhance the transferability and overall robustness of our findings. Second, the subgroup of participants classified as having preregistration experience was heterogeneous in terms of the type and extent of their experiences. While most had directly preregistered an animal study, some had partial or related experience with preregistration. As recruitment of researchers with direct preregistration experience proved particularly challenging, we opted to include all types of preregistration experience in the experienced subgroup, including partial or related experience. This heterogeneity should be considered when interpreting findings related to preregistration experience. Third, researcher bias may have influenced data collection, as the interviewer had prior experience with preregistration and was generally supportive of the practice. To minimize this bias, peer debriefing was employed. The interviewer (C.P.) engaged in regular discussions with a second researcher (S.M.L.), who led the transcription process and did not have prior preregistration experience, as well as with the study supervisor (H.W.), to critically reflect on potential preconceptions and ensure transparency. Fourth, interpretation bias might have influenced the analysis, as qualitative approaches involve a certain degree of subjectivity. To address this issue, the interviewer (C.P.) and a further researcher (G.S.) independently coded the interview transcripts and collaboratively conducted the thematic analysis to reduce subjective interpretation and enhance the credibility of the findings.

## 5. Conclusion

Preregistration remains largely uncommon and underused within animal research. While researchers with previous preregistration experience tend to recognize its value, those who have not yet engaged in it remain hesitant and often unconvinced of its benefits. Increasing awareness, addressing misconceptions and providing targeted training and institutional support appear to be crucial for its wider adoption. In addition, simplifying procedures and linking preregistration to the existing authorization procedure may help reduce administrative barriers and encourage broader engagement. Nevertheless, our findings show that preregistration should remain voluntary and incentive-based to ensure acceptance and avoid opposition from the research community.

## Supplementary Material



## Data Availability

Anonymized transcripts from participants who consented to their publication are available in the University of Bern data repository BORIS at the following DOI: https://doi.org/10.48620/92462. Supplementary material is available online [58].
