# Supplementary material for: Qualitative study reveals perceived barriers and facilitators of preregistration in animal research
Source: R Soc Open Sci. 2026 Oct 28;13(10):261156. doi: 10.1098/rsos.261156 (PMC13714398; doi:10.1098/rsos.261156)
Supplement: Supplementary file 1 [file rsos.261156.zip › rsos.261156/rsos261156_si_001.pdf]

Electronic Supplementary Material

Title: Qualitative study reveals perceived barriers and facilitators of preregistration in animal research

Authors: Cristina Priboi, Simone Lalor, Evie Vergauwe, Bernice Elger, Hanno Würbel

Journal: *Royal Society Open Science*

Article DOI: https://doi.org/10.1098/rsos.261156

Table S1. Deviations from preregistration plan

**Unregistered Steps**					
	**Details**		**Original Wording**	**Unregistered Step Description**	**Reader Impact**
1	Type	Sample	If the targeted number of participants is not reached, the snowballing technique and purposive sampling will be applied to expand the sample.	After using the snowballing technique (asking interview participants to recommend others with preregistration experience) and purposive sampling (searching registries for animal studies to identify potential participants), an unplanned additional recruitment method was employed. This step was necessary because the planned strategies did not yield a sufficient number of animal researchers with preregistration experience. Consequently, the Federal Food Safety and Veterinary Office (FSVO) sent an invitation email to participants who had completed the online questionnaire for the quantitative component of the mixed-methods project, of which the current qualitative study is a part. In this email, FSVO informed animal researchers about the ongoing qualitative study and the possibility of participating. Before implementing this additional recruitment step, the Ethical Commission of the Faculty of Psychology at the University of Bern was notified.	This unplanned step does not affect the reader's interpretation of the study, as the FSVO email led to the identification of 20 additional animal researchers, none of whom had preregistration experience. Therefore, no new participants were recruited through this pathway.
	Timing	During data collection			

Table S2. Standards for Reporting Qualitative Research (SRQR)

Title and abstract

	Page/line no(s).
**Title** - Concise description of the nature and topic of the study Identifying the study as qualitative or indicating the approach (e.g., ethnography, grounded theory) or data collection methods (e.g., interview, focus group) is recommended	p. 1, lines 4-5
**Abstract** - Summary of key elements of the study using the abstract format of the intended publication; typically includes background, purpose, methods, results, and conclusions	p. 2, lines 25-44

Introduction

**Problem formulation** - Description and significance of the problem/phenomenon studied; review of relevant theory and empirical work; problem statement	p. 3, lines 49-85
**Purpose or research questio**n - Purpose of the study and specific objectives or questions	pp. 3-4, lines 85-92

Methods

**Qualitative approach and research paradigm** - Qualitative approach (e.g., ethnography, grounded theory, case study, phenomenology, narrative research) and guiding theory if appropriate; identifying the research paradigm (e.g., postpositivist, constructivist/ interpretivist) is also recommended; rationale**	p. 4, lines 105-115
**Researcher characteristics and reflexivity** - Researchers' characteristics that may influence the research, including personal attributes, qualifications/experience, relationship with participants, assumptions, and/or presuppositions; potential or actual interaction between researchers' characteristics and the research questions, approach, methods, results, and/or transferability	p. 4, lines 116-127
**Context** - Setting/site and salient contextual factors; rationale**	p. 5, lines 128-146
**Sampling strategy** - How and why research participants, documents, or events were selected; criteria for deciding when no further sampling was necessary (e.g., sampling saturation); rationale**	pp. 5-6, lines 147-208<br>Figure S3<br>Figure S4
**Ethical issues pertaining to human subjects** - Documentation of approval by an appropriate ethics review board and participant consent, or explanation for lack thereof; other confidentiality and data security issues	p. 7, lines 209-221<br>p. 22, lines 741-744
**Data collection methods** - Types of data collected; details of data collection procedures including (as appropriate) start and stop dates of data collection and analysis, iterative process, triangulation of sources/methods, and modification of procedures in response to evolving study findings; rationale**	p. 7, lines 223-231

**Data collection instruments and technologies** - Description of instruments (e.g., interview guides, questionnaires) and devices (e.g., audio recorders) used for data collection; if/how the instrument(s) changed over the course of the study	p. 7-9, lines 232-244<br>Table 1
**Units of study** - Number and relevant characteristics of participants, documents, or events included in the study; level of participation (could be reported in results)	p. 9, lines 245-249<br>pp. 10-12, lines 304-346<br>Table 2
**Data processing** - Methods for processing data prior to and during analysis, including transcription, data entry, data management and security, verification of data integrity, data coding, and anonymization/de-identification of excerpts	p. 7, lines 228-231<br>p. 9, lines 250-254
**Data analysis** - Process by which inferences, themes, etc., were identified and developed, including the researchers involved in data analysis; usually references a specific paradigm or approach; rationale**	pp. 9-10, lines 255-282
**Techniques to enhance trustworthiness** - Techniques to enhance trustworthiness and credibility of data analysis (e.g., member checking, audit trail, triangulation); rationale**	p. 10, lines 283-302

Results/findings

**Synthesis and interpretation** - Main findings (e.g., interpretations, inferences, and themes); might include development of a theory or model, or integration with prior research or theory	pp. 12-17, lines 347-547
**Links to empirical data** - Evidence (e.g., quotes, field notes, text excerpts, photographs) to substantiate analytic findings	pp. 12-17, lines 353-547<br>Figures 1-6<br>Table S8<br>Table S9

Discussion

**Integration with prior work, implications, transferability, and contribution(s) to the field -** Short summary of main findings; explanation of how findings and conclusions connect to, support, elaborate on, or challenge conclusions of earlier scholarship; discussion of scope of application/generalizability; identification of unique contribution(s) to scholarship in a discipline or field	pp. 17-20, lines 548-671
**Limitations** - Trustworthiness and limitations of findings	pp. 20-21, lines 672-705

Other

**Conflicts of interest** - Potential sources of influence or perceived influence on study conduct and conclusions; how these were managed	p. 23, lines 753–754
**Funding** - Sources of funding and other support; role of funders in data collection, interpretation, and reporting	p. 22, lines 729–731

Figure S3: Targeted study sample

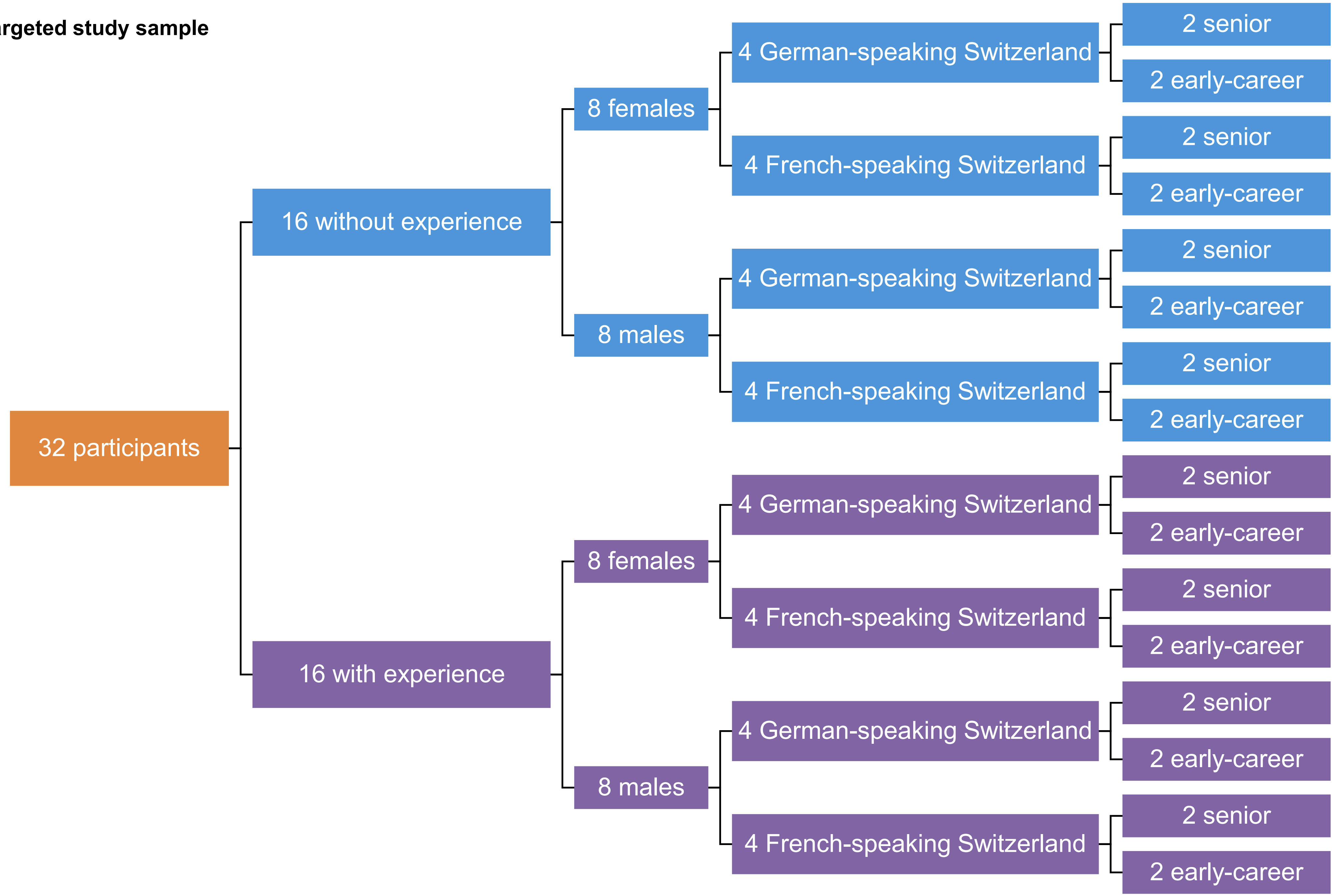

Figure S4: Final study sample

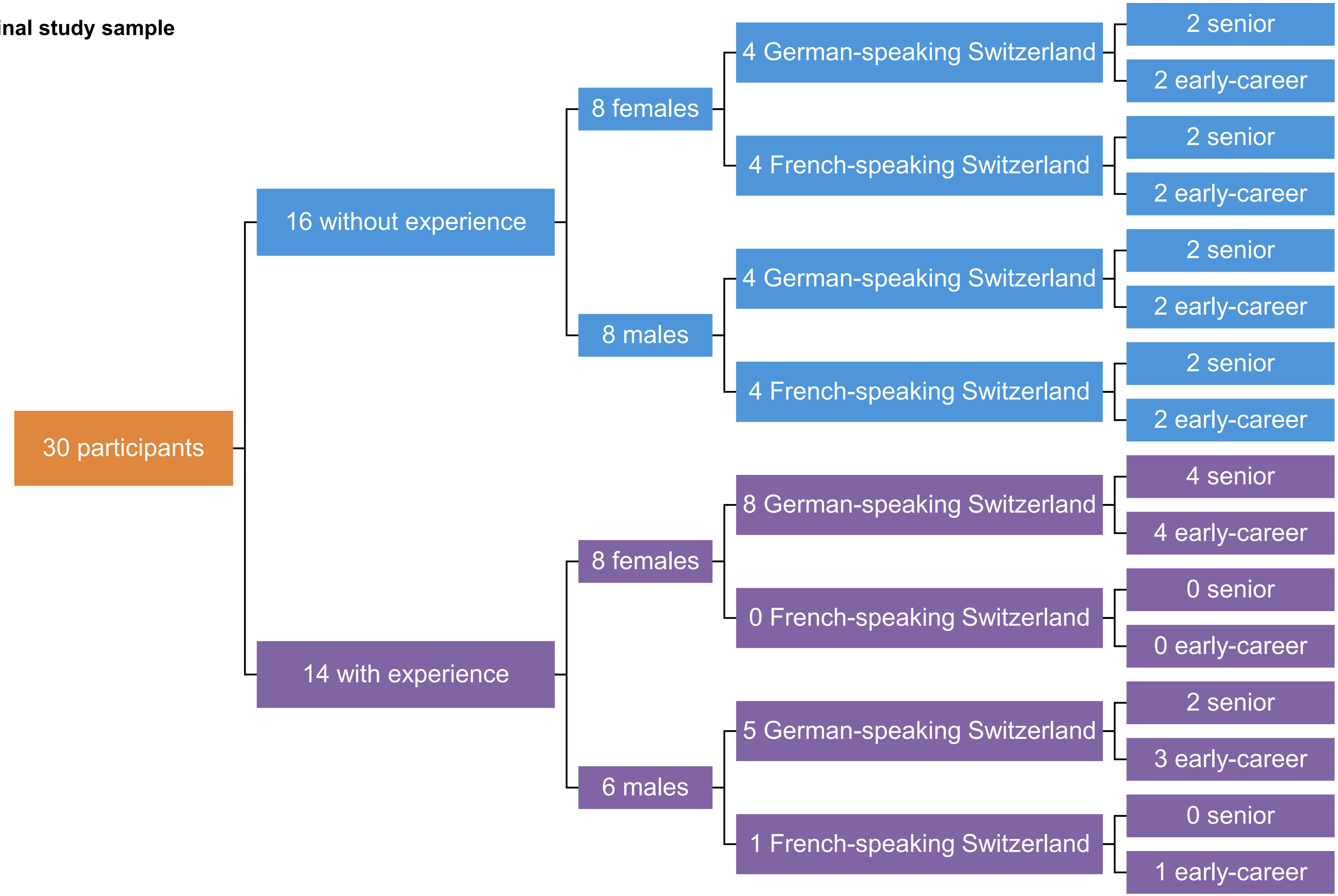

b
UNIVERSITÄT
BERN

Participant Information Letter

1. Research project title: *Preregistration in Animal Experimentation – A Feasibility Study*

2. Principal investigators

Prof. Dr. Hanno Würbel
Head of the Animal Welfare Division
Veterinary Public Health Institute
University of Bern
Switzerland
E-mail: hanno.wuerbel@unibe.ch
Tel.: +41 31 684 25 30

Assoc. Prof. Dr. Evie Vergauwe
Geneva University Neurocenter
Faculty of Psychology and Educational Sciences
University of Geneva
Switzerland
E-mail: evie.vergauwe@unige.ch
Tel.: +41 22 379 09 15

3. Project collaborators

Prof. Dr. Bernice Elger
Head of the Institute for Biomedical Ethics
Faculty of Medicine
University of Basel
Switzerland
E-mail: b.elger@unibas.ch
Tel.: +41 61 207 17 78

Dr. Boris Mayer
Lecturer for Research Methods
Institute of Psychology
University of Bern
Switzerland
E-mail: boris.mayer@unibe.ch
Tel.: +41 31 684 51 94

Dr. Cristina Priboi
Postdoctoral researcher
Animal Welfare Division
Veterinary Public Health Institute
University of Bern
Switzerland
E-mail: cristina.priboi@unibe.ch
Tel.: +41 31 684 24 28

4. Who are we and why are we conducting this study?

We are a multidisciplinary team aiming to investigate scientists' attitudes and potential concerns regarding study preregistration in animal experimentation. Our team is primarily based at the Animal Welfare Division of the Veterinary Public Health Institute at the University of Bern. This study is funded by the Federal Food Safety and Veterinary Office (FSVO) and part of a larger research project on open research data (SIRRO) supported by swissuniversities. The shift towards Open and Reproducible Science in the scientific community seeks to enhance rigor, transparency, and reproducibility in research, thereby ensuring credibility and building trust. Preregistration, defined as the a priori registration of hypotheses, study designs and analysis plans in open repositories, stands as one of the most challenging yet potentially most transformative practices in Open and Reproducible Science. However, there is a risk associated with changing policies either too extensively or too prematurely, without or before fully understanding their potential benefits, costs, and practical implications. Thus, our study aims to assess the strengths, weaknesses, opportunities, and threats related to preregistration in animal research. The findings of our research will offer valuable insights into the applicability of preregistration to animal research and could inform future policy decisions and infrastructure development when implementing preregistration of animal research in Switzerland.

5. Why have you been invited to take part in this study?

You have been contacted for this study because you are registered as a study director on the Animex-ch platform and you have indicated that you are willing to participate in an interview. Your own perspective as an expert in the animal research field is what we are looking for in this study.

6. Do you have to take part to this study?

Your participation is entirely voluntary, and you are under no obligation to take part in this study. The decision to participate or not will be kept strictly confidential. Should you choose to participate, you will need to provide formal agreement by signing this consent form. Even after giving consent and participating in the interview, you will have the option to stop the interview at any time and/or withdraw your data up to two weeks after the interview without having to provide any justification. To withdraw, simply contact the interviewer via email and inform them of your decision. In such an event, the original recording of your interview and any related documents will be promptly destroyed.

7. What data will be collected and how long will it take?

The research method used in this study is a semi-structured interview. This will allow the research team to explore participants' thoughts, feelings, and beliefs on the specific topic through personal questions and discussions. It provides participants with the freedom to express their views in their own words, fostering a non-judgmental environment. As a participant, you will be required to:

- Attend the scheduled interview, which you have agreed to participate in. Your interview will last approximately 60 minutes.
- Understand that there are no right or wrong answers – all opinions or statements will be valued and analyzed in an anonymized way.
- Consent to having the interview discussion audio-recorded and transcribed verbatim for further analysis. The transcripts will be anonymized to ensure your privacy, making it impossible to identify you at any stage. These recordings will be securely deleted at the end of the project unless you request to withdraw from the study (refer to section 3 above). Additionally, notes may be taken during the interviews to assist in the analysis of the transcripts.

8. What will happen with the data of this study?

The recordings will be stored in an encrypted format, labeled with participants' IDs, to prevent the identification of specific participants while still allowing the study team to access them again if necessary. The audio recordings of the interviews will be transcribed verbatim, but all information that could lead to your identification (such as names, workplaces, or other identifying details) will be omitted. Your name or any other identifying information will never appear on the internet, in any publication, or in any report. Data will be securely stored on a server at the University of Bern, with access granted only to study personnel. All persons who have access to participants' data within the framework of the project are bound by confidentiality obligations. The audio recordings will be destroyed after the last article of the study has been accepted for publication. Your data will be combined with data from other participants for analyses that will be reported in scientific publications. Upon completion of the study, your anonymized interview transcription will be stored in an open-access data repository. Demographic variables such as age, educational level, official research position, and field of research which when combined could lead to your identification, will be anonymized by binning before sharing on online open-access data repositories. In the event of the publication of this study's findings, the research team may receive requests from external researchers for access to your demographic data. If such requests are made, they will be thoroughly reviewed by the research team, and data might be shared with other researchers. Furthermore, external researchers will be required to adhere to the same data security standards as this project.

9. What are the possible risks and benefits of your participation?

Participation in this study poses no anticipated risks and the interviews are expected to cause no disruptions to your daily life. While there is no direct benefit to you from participating in this study, your involvement will

be a valuable contribution to research. Your answers will offer valuable insights into the pros and cons of study preregistration, potentially guiding future policymaking and infrastructure development for implementing preregistration of animal experiments in Switzerland.

10. Who has reviewed and approved this study?

This study has been reviewed and received ethical approval from the Ethics Committee of the Faculty of Psychology at the University of Bern (Nr. 2024-03-06).

11. Who should you contact for further information?

Dr. Cristina Priboi, E-Mail: cristina.priboi@unibe.ch, Tel.: +41 31 684 24 28

Interview Consent Form

Two copies of this signed consent form will be produced: one for your personal records and the other to be securely stored in a locked cabinet at the University of Bern. No copies will be made from the latter, and only authorized personnel will have access to it.

By signing this form, I agree to the following statements:

- I have read and understood the information sheet about the research project. I have had the opportunity to consider this information and make an informed decision.
- I have not been coerced into participating in this study.
- I am aware that I can withdraw my data within two weeks after my participation in the interviews, without providing any reason and without any negative repercussions.
- I am aware that I can withdraw my data within two weeks after my participation in the interviews, without providing any reason and without any negative repercussions.
- All information I provide will be used for research purposes only.
- Upon completion of the study, my anonymized interview transcript will be stored in an open-access data repository.
- In the event of requests from external researchers for access to my data, these requests will be thoroughly reviewed by the research team. Additionally, external researchers will be required to adhere to the same data security standards as this project in order to gain access to my information.

Do you consent to participate in this interview?

☐ **I consent** ☐ **I do not consent**

Do you consent to having your anonymized data included in an open-access data repository?

☐ **I consent** ☐ **I do not consent**

Name of Participant	Date	Signature
Name of Interviewer	Date	Signature

Interview Consent Form

By signing this form, I agree to the following statements:

- I have read and understood the information sheet about the research project. I have had the opportunity to consider this information and make an informed decision.
- I have not been coerced into participating in this study.
- I am aware that I can withdraw my data within two weeks after my participation in the interviews, without providing any reason and without any negative repercussions.
- I am aware that I can withdraw my data within two weeks after my participation in the interviews, without providing any reason and without any negative repercussions.
- All information I provide will be used for research purposes only.
- Upon completion of the study, my anonymized interview transcript will be stored in an open-access data repository.
- In the event of requests from external researchers for access to my data, these requests will be thoroughly reviewed by the research team. Additionally, external researchers will be required to adhere to the same data security standards as this project in order to gain access to my information.

Do you consent to participate in this interview?

☐ I consent **☐ I do not consent**

Do you consent to having your anonymized data included in an open-access data repository?

☐ I consent **☐ I do not consent**

Name of Participant	Date	Signature
Name of Interviewer	Date	Signature

Consent Form S6. Consent form for online participation

UNIVERSITÄT BERN

Participant Information Letter

1. Research project title: *Preregistration in Animal Experimentation – A Feasibility Study*

2. Principal investigators

Prof. Dr. Hanno Würbel
Head of the Animal Welfare Division
Veterinary Public Health Institute
University of Bern
Switzerland
E-mail: hanno.wuerbel@unibe.ch
Tel.: +41 31 684 25 30

Assoc. Prof. Dr. Evie Vergauwe
Geneva University Neurocenter
Faculty of Psychology and Educational Sciences
University of Geneva
Switzerland
E-mail: evie.vergauwe@unige.ch
Tel.: +41 22 379 09 15

3. Project collaborators

Prof. Dr. Bernice Elger
Head of the Institute for Biomedical Ethics
Faculty of Medicine
University of Basel
Switzerland
E-mail: b.elger@unibas.ch
Tel.: +41 61 207 17 78

Dr. Boris Mayer
Lecturer for Research Methods
Institute of Psychology
University of Bern
Switzerland
E-mail: boris.mayer@unibe.ch
Tel.: +41 31 684 51 94

Dr. Cristina Priboi
Postdoctoral researcher
Animal Welfare Division
Veterinary Public Health Institute
University of Bern
Switzerland
E-mail: cristina.priboi@unibe.ch
Tel.: +41 31 684 24 28

4. Who are we and why are we conducting this study?

We are a multidisciplinary team aiming to investigate scientists' attitudes and potential concerns regarding study preregistration in animal experimentation. Our team is primarily based at the Animal Welfare Division of the Veterinary Public Health Institute at the University of Bern. This study is funded by the Federal Food Safety and Veterinary Office (FSVO) and part of a larger research project on open research data (SIRRO) supported by swissuniversities. The shift towards Open and Reproducible Science in the scientific community seeks to enhance rigor, transparency, and reproducibility in research, thereby ensuring credibility and building trust. Preregistration, defined as the a priori registration of hypotheses, study designs and analysis plans in open repositories, stands as one of the most challenging yet potentially most transformative practices in Open and Reproducible Science. However, there is a risk associated with changing policies either too extensively or too prematurely, without or before fully understanding their potential benefits, costs, and practical implications. Thus, our study aims to assess the strengths, weaknesses, opportunities, and threats related to preregistration in animal research. The findings of our research will offer valuable insights into the applicability of preregistration to animal research and could inform future policy decisions and infrastructure development when implementing preregistration of animal research in Switzerland.

5. Why have you been invited to take part in this study?

You have been contacted for this study because you are registered as a study director on the Animex-ch platform and you have indicated that you are willing to participate in an interview. Your own perspective as an expert in the animal research field is what we are looking for in this study.

6. Do you have to take part to this study?

Your participation is entirely voluntary, and you are under no obligation to take part in this study. The decision to participate or not will be kept strictly confidential. Should you choose to participate, you will need to provide formal agreement by signing this consent form. Even after giving consent and participating in the interview, you will have the option to stop the interview at any time and/or withdraw your data up to two weeks after the interview without having to provide any justification. To withdraw, simply contact the interviewer via email and inform them of your decision. In such an event, the original recording of your interview and any related documents will be promptly destroyed.

7. What data will be collected and how long will it take?

The research method used in this study is a semi-structured interview. This will allow the research team to explore participants' thoughts, feelings, and beliefs on the specific topic through personal questions and discussions. It provides participants with the freedom to express their views in their own words, fostering a non-judgmental environment. As a participant, you will be required to:

- Attend the scheduled interview, which you have agreed to participate in. Your interview will last approximately 60 minutes.
- Understand that there are no right or wrong answers – all opinions or statements will be valued and analyzed in an anonymized way.
- Consent to having the interview discussion audio-recorded and transcribed verbatim for further analysis. The transcripts will be anonymized to ensure your privacy, making it impossible to identify you at any stage. These recordings will be securely deleted at the end of the project unless you request to withdraw from the study (refer to section 3 above). Additionally, notes may be taken during the interviews to assist in the analysis of the transcripts.

8. What will happen with the data of this study?

The recordings will be stored in an encrypted format, labeled with participants' IDs, to prevent the identification of specific participants while still allowing the study team to access them again if necessary. The audio recordings of the interviews will be transcribed verbatim, but all information that could lead to your identification (such as names, workplaces, or other identifying details) will be omitted. Your name or any other identifying information will never appear on the internet, in any publication, or in any report. Data will be securely stored on a server at the University of Bern, with access granted only to study personnel. All persons who have access to participants' data within the framework of the project are bound by confidentiality obligations. The audio recordings will be destroyed after the last article of the study has been accepted for publication. Your data will be combined with data from other participants for analyses that will be reported in scientific publications. Upon completion of the study, your anonymized interview transcription will be stored in an open-access data repository. Demographic variables such as age, educational level, official research position, and field of research which when combined could lead to your identification, will be anonymized by binning before sharing on online open-access data repositories. In the event of the publication of this study's findings, the research team may receive requests from external researchers for access to your demographic data. If such requests are made, they will be thoroughly reviewed by the research team, and data might be shared with other researchers. Furthermore, external researchers will be required to adhere to the same data security standards as this project.

9. What are the possible risks and benefits of your participation?

Participation in this study poses no anticipated risks and the interviews are expected to cause no disruptions to your daily life. While there is no direct benefit to you from participating in this study, your involvement will

be a valuable contribution to research. Your answers will offer valuable insights into the pros and cons of study preregistration, potentially guiding future policymaking and infrastructure development for implementing preregistration of animal experiments in Switzerland.

10. Who has reviewed and approved this study?

This study has been reviewed and received ethical approval from the Ethics Committee of the Faculty of Psychology at the University of Bern (Nr. 2024-03-06).

11. Who should you contact for further information?

Dr. Cristina Priboi, E-Mail: cristina.priboi@unibe.ch, Tel.: +41 31 684 24 28

Interview Consent Form

Two copies of this signed consent form will be produced: one for your personal records and the other to be securely stored in a locked cabinet at the University of Bern. No copies will be made from the latter, and only authorized personnel will have access to it.

By signing this form, I agree to the following statements:

- I have read and understood the information sheet about the research project. I have had the opportunity to consider this information and make an informed decision.
- I have not been coerced into participating in this study.
- I am aware that I can withdraw my data within two weeks after my participation in the interviews, without providing any reason and without any negative repercussions.
- I am aware that I can withdraw my data within two weeks after my participation in the interviews, without providing any reason and without any negative repercussions.
- All information I provide will be used for research purposes only.
- Upon completion of the study, my anonymized interview transcript will be stored in an open-access data repository.
- In the event of requests from external researchers for access to my data, these requests will be thoroughly reviewed by the research team. Additionally, external researchers will be required to adhere to the same data security standards as this project in order to gain access to my information.

Do you consent to participate in this interview?

☐ **I consent** ☐ **I do not consent**

Do you consent to having your anonymized data included in an open-access data repository?

☐ **I consent** ☐ **I do not consent**

Name of Participant	Date	Signature
Name of Interviewer	Date	Signature

Definition S7: Definition of preregistration

- Study preregistration is a distinct process from the registration of applications for animal experiments on Animex-ch [web application to record licenses for animal experimentation in Switzerland].
- Preregistration involves registering the study protocol on an independent repository before data collection or data analysis.
- The repositories can be general or field specific. Here are some examples of available repositories:
  - animalstudyregistry.org
  - osf.io (Open Science Framework)
  - ClinicalTrials.gov
  - AsPredicted.org
- These repositories offer templates for study preregistration.
- Upon registration, a time-stamped record and a DOI of the study protocol is generated.
- The preregistered record can be published immediately or after an embargo period.
- Deviations from the registered study protocol must be noted in a new record or in the published manuscript.
- The goal of preregistration is to ensure transparency in research.

Table S8. Coding system – Coder CP

Codes and subcodes	Frequency
**Overall experience**	
Preregistration experience	
Preregistered before	9
Preferred registry	2
No encountered problems	12
Never heard	6
Never preregistered	14
Not aware it exists (for animals)	5
Underused by other	19
Not known by other	6
Source	
Research institution / during studies	4
Supervisor	3
Clinal trials	5
Survey (never heard before)	7
Colleagues	5
Readings	2
Further education	6
Scientific exchange/meeting/conference	5
Definition	
Correct definition	27
Incomplete/misunderstanding	7
**General views**	
Perception - self	
Beneficial - self	15
Neutral perception - self	6
Problematic - self	8
Perception - others	
Neutral perception - others	2
Positive perception - others	5

Negative perception - others	21
Misconceptions	
Miss - Confirmatory vs. exploratory	4
Miss - External complains / harassment	1
Miss - Lack of flexibility	8
Miss - Scooping	3
"Just trust me"	5
"Older" scientists	6
**Suggestions**	
Clear demonstration of benefits (for all types of research)	28
Easy-to-use tool / clear (or flexible) template	12
Endorsement / Requirement	2
Endorsement FSVO	2
Endorsement from institutions	10
Endorsement from scientific community	4
Endorsement from funders	12
Endorsement from journals	14
Endorsement from animal welfare-related bodies	1
Harmonization of guidelines	11
Incentives / Benefits	14
Job benefits	1
Institutional incentives	6
Faster authorization process	4
Funding agencies benefits	4
Publishing benefits	8
Increase embargo	5
Increase knowledge / awareness	15
Info on where and how to preregister	5
Provide examples	1
Workshops / courses / continued education	13
International implementation, not only CH	13
Link with Animex / Form A	25

Open license number	8
ChatGPT/AI	2
Use an existing platform / no new Swiss registry	3
Offer support & resources - dedicated staff, funds, AWO etc.	25
University ethics commission / internal preregistration	8
Strong confidentiality measures	1
Voluntary rather than mandatory	24
Stepwise mandatory	4
If mandators, only for some (confirmatory) research	4
Make it mandatory	12
Negative reaction from community	11
If mandatory	16
**Positive aspects (facilitators)**	
Accountability	9
Better consideration of animal use (3R) / animal welfare	14
Better (study) planning	29
Better public / external understanding	7
Better statistical analysis / Prevents ORPs	14
Prevents cherry picking	6
Prevents HARKing	9
Prevents p-hacking	2
Better study design / thorough methods	10
Done by others / research community	3
Encourages scientific feedback & exchange	3
Facilitates collaborations & synergies	6
Helps with drafting papers / Helps with publishing	4
Higher research quality	21
Improves reproducibility / reliability	12
Improves time & project management	3
Increases credibility	3
Increases trustworthiness	1
Less deviations from protocols / experiments	12

More transparency	30
Open Science support	1
Prevents publication bias / all data is known	25
Reduces parallel research	13
Protects intellectual property	3
Provides a track record	3
Reduces researcher bias	4
More benefits for some type of studies (e.g. confirmatory)	16
**Negative aspects (barriers)**	
Blocks innovation	3
Bureaucratic/administrative burden/extra work	62
Can be faked	5
Causes delays	5
Embargo period too long/short	2
External complaints / harassment	8
Increases (research) costs	1
Internal preregistration	3
Lack of flexibility	28
Lack of knowledge	4
Lack of resources	2
No connection to Animex	2
No incentives	1
Nobody checks the preregistration forms / No feedback	14
Not (only) my decision	1
Not commonly established	4
Not compatible with industry	15
Not possible due to confidentiality agreements	5
Not feasible (but ideal or fantasy)	5
Not interested / not necessary	2
Not requested by journals, funders, institutions etc.	1
Not suitable for my (exploratory/fundamental) research	37
Overregulation in Switzerland	8

	Reduces international competitivity	3
	Reduction in the number on studies / animal research	5
	Redundant - e.g. Animex / grants	23
	Scooping / intellectual property	36
	Time consuming	25
	Too many deviations / branching / unpredictable	26
	Unaware of the benefits and impact	7
**OTHER**		
	Platforms	6
	Future	31
	Mindset/personal ethic	3

Table S9. Coding system – Coder GS

Codes and subcodes	Frequency
**Overall experience**	
Preregistration Experience	
Never preregistered	16
Preregistered before	15
Definition	
correct definition	24
incomplete/misunderstanding	9
Source	
Survey (never heard before)	6
Colleagues	5
Readings	4
Conferences	3
Further education	9
Scientific exchange	8
Research	3
**General views**	
Perception - self	
Beneficial	6
Neutral Perception	9
Problematic	4
Perception - others	
Positive perception - others	1
Neutral Perception - others	2
Negative Perception- others	12
Underused by others	7
Not known by others	5
**Suggestions**	
Badges for PreReg studies	
Clear demonstration of benefits (for all types of research)	29

Easy-to-use tool / clear (or flexible) template	17
Endorsement / Requirement	3
Endorsement from PIs	1
Endorsement from institutions	14
Endorsement from scientific community	3
Endorsement from funders	12
Endorsement from (good) journals	18
Endorsement from animal welfare-related bodies	6
Flexibility - possibility to make changes	2
Harmonization of guidelines	12
If mandators, only for confirmatory research	3
Stepwise mandatory	2
Make it mandatory	23
More guidance	4
Incentives / Benefits	9
Institutional incentives	3
Faster authorization process	5
Funding agencies benefits	2
Publishing benefits	6
Increase embargo	4
Increase knowledge / awareness	13
Info on where and how to preregister	10
Provide examples	3
Workshops / courses / continued education	11
International implementation, not only CH	4
Link with Animex / Form A	25
Offer expert review of PreReg forms	3
Offer support & resources - dedicated staff, funds etc.	27
Strong confidentiality measures	1
Voluntary rather than mandatory	12
**Positive aspects (facilitators)**	
Archive	1

Better consideration of animal use (3R)	14
Better (study) planning	39
Better public / external understanding	11
Better statistical analysis	8
Prevents HARKing	7
Prevents p-hacking	6
Better study design / thorough methods	15
Compliance with original study plan	19
Encourages scientific feedback & exchange	8
Facilitates collaborations & synergies	6
Good research practices / prevention of questionable research practices	30
Helps with drafting papers / Speeds up the journal review process	2
Higher research quality	27
Improves reproducibility / reliability	16
Improves time & project management	7
Increases credibility	4
Increases trustworthiness	7
Less deviations	2
More transparency	35
Open Science support	10
Prevents publication bias / all data is known	22
Protects intellectual property	1
Provides a track record	5
Reduces parallel research	14
Reduces researcher bias	4
Uniformity of protocols / experiments	0
More benefits for confirmatory studies than exploratory / fundamental research	7
No benefits	24
**Negative aspects (barriers)**	
Blocks innovation	15

Bureaucratic/administrative burden	79
Causes delays	14
Embargo period too long	4
External complaints / harassment	12
Increases (research) costs	7
Internal preregistration	1
Lack of flexibility	45
Lack of guidance	5
Lack of harmonization	2
Lack of knowledge	21
Lack of resources	5
Loss of creativity	2
No connection to Animex	1
No incentives	2
No support from research partners / co-authors	1
Nobody checks the preregistration forms / No feedback	14
Not (only) my decision	1
Not commonly established	17
Not compatible with industry	11
Not compatible with patent systems	1
Not Foolproof	2
Not interested / not necessary	8
Not possible due to confidentiality agreements	11
Not requested by journals, funders, institutions etc.	5
Not suitable for my (exploratory/fundamental) research	35
Overregulation in Switzerland	11
Preregistered but not published	2
Reduces international competitivity	6
Reduction in the number on studies / animal research	8
Redundant - e.g. Animex	22
Scooping	39

Time consuming	34
Too many deviations	36
Unaware of the benefits and impact	13
Relocation to other countries	0
